# Enhancement of Bioactive Constituents in Fresh Cauliflower By-Products in Challenging Climate Conditions

**DOI:** 10.3390/antiox11050958

**Published:** 2022-05-12

**Authors:** Jacinta Collado-González, María Carmen Piñero, Ginés Otalora, Josefa López-Marín, Francisco M. del Amor

**Affiliations:** Department of Crop Production and Agri-Technology, Murcia Institute of Agri-Food Research and Development (IMIDA), C/Mayor s/n, 30150 Murcia, Spain; mariac.pinero2@carm.es (M.C.P.); gines.oralora@carm.es (G.O.); josefa.lopez38@carm.es (J.L.-M.); franciscom.delamor@carm.es (F.M.d.A.)

**Keywords:** healthier cauliflower by-product, climate change, health-promoting compounds, sustainable strategy

## Abstract

In order to mitigate the detrimental impact that climate change is having on plants, the study of new practices that allow for the reduction of such effects has become imperative. In addition, the revaluation of the promotion of healthy plant by-products has also markedly increased in importance in recent years. In this work, the modifications in biomass and some antioxidant compounds of cauliflower by-products treated with putrescine under extreme temperatures in two different CO_2_ scenarios (the control (400 ppm) and a high concentration of CO_2_ (1000 ppm)) were studied. Additionally, the compositions of inner and outer leaves were also compared. According to results found in this work, cauliflower grown under elevated CO_2_ and treated with putrescine (2.5 mM) prior to heat stress showed the highest biomass accumulation (20%) compared to the control. Moreover, in the outer leaves from cauliflower grown under elevated CO_2_ and treated with putrescine prior to high temperature exposure, the highest biosynthesis of sugars (20%) was recorded. Although cauliflower by-products turned out to be rich in polyamines (208.6 nmoles g^−1^ fresh weight (FW) and 124.3 nmoles g^−1^ FW for outer and inner leaves, respectively) and phenolic compounds (1070.2 mg gallic acid equivalents ( (GAE) 100 g^−1^ FW in outer leaves and 772.0 mg GAE 100 g^−1^ FW in inner leaves), it was the outer leaves that after applying the new strategy showed the greatest increase in polyamines (68%) and phenolic compounds (39%), obtaining here the highest increase in antioxidant activity (3%). Thus, they should no longer be regarded as mere by-products and should be used for pharmaceutical or nutraceutical purposes. The novel strategy presented in this work may allow us to take advantage of both the continued increase in CO_2_ and heat waves that are becoming more frequent.

## 1. Introduction

Climate change is an irreversible threat that will have a considerable negative effect on agriculture, compromising the food safety of the general population [1]. Over the last few years, there has been growing concern about the effects of climate change on crop production [2].

To our knowledge, there is limited information about the effect of extreme heat and high CO_2_ level stress combinations on plants [3,4]. Heat stress can alter the normal growth and development of plants [5]. Some authors have reported that a high level of CO_2_, in addition to stimulating biomass production, also has a dampening effect on the negative effects of abiotic stress on plants, such as ozone, heat, drought, nutrient imbalance, and ion toxicity [3,6,7].

Cauliflower is a frequently consumed vegetable that belongs to the *Brassica* species [8]. In fact, cauliflower is considered as one of the most important Brassicas due to its healthy properties. These health benefits are due to the fact that this vegetable contains a substantial amount of dietary fiber, and a substantial number of vitamins; minerals; and bioactive compounds, such as glucosinolates, isothiocyanates, and polyamines [9,10]. These health-promoting compounds have been found to be effective in protecting against many chronic human diseases, including several kinds of cancer [8].

Polyamines (PAs), including putrescine (Put), spermidine (Spd), spermine (Spm), and cadaverine (Cad), are a group of essential growth regulators in plants. They have been used to improve the tolerance of plants under different conditions of abiotic stress. Specifically, the exogenous application of spermidine to cauliflower was shown to confer some protection to cauliflower against heat stresses. Collado-González et al. [9] found that plants treated with 4 mM Spd prior to being subjected to heat stress showed an increase in their content of anions (203%), total phenolic compounds (23%), antioxidant activity (27%), total sugars (43%), and PAs (344%).

As mentioned, PAs are also important for living a healthy life. They play an important role in protecting adults from age-associated metabolic disorders and can help to improve their fertility functions [11].

On the other hand, in order to help mitigate climate change, an awareness of the need to obtain more sustainable crops has also been observed in recent years [12]. *Brassica oleracea* crops such as cauliflower and broccoli are widely grown worldwide, with the Region of Murcia being the largest producer of these crucifers in Europe. The consumption of these crucifers has greatly increased in recent years due to their nutritional, nutraceutical, and anticancer properties [13]. However, it is important to be aware that a large number of these plants are directly classified as residues, and more than 50% of this by-product is leaf [14]. The wasting of this material has a negative impact on the environment [15]. 

Therefore, the main goal of this work was to explore the effect of high temperature and elevated CO_2_ on the content of bioactive compounds such as PAs, total phenols, antioxidant activity, and sugars in the cauliflower cv “Moonshine” by-products. All of these parameters are of paramount importance for human health. The main objectives of this work were: (i) to explore the possible synergistic effect between heat stress and elevated CO_2_ in order to propose a new sustainable strategy for cauliflower cv Moonshine. and (ii) to compare the nutritional quality found in the external and internal leaves. The results of this study may provide new insights into the potential revaluation of cauliflower by-products.

## 2. Materials and Methods

### 2.1. Plant Material and Growth Conditions

Cauliflower plants (*Brassica oleracea* var. botrytis L.), cv. Moonshine, were obtained from a commercial nursery (Enza Zaden España S.L., Almería, Spain) 30 days after germination, and seedlings of a similar size were selected. Plants were grown in 5 L black containers filled with coconut coir fiber (Pelemix, Alhama de Murcia, Murcia, Spain). For this experiment, eight treatments were studied, using five plants per treatment (forty plants in total). All these plants were irrigated with a modified Hoagland’ solution (mM): NO_3_^−^: 12.0; H_2_PO_4_^−^: 1.0; SO_4_^2−^: 3.5; K^+^: 7.0; Ca^2+^: 4.5; Mg^2+^: 2.0. The experiment was performed in a climate-controlled chamber designed by our department specifically for plant research [16]. This chamber allowed us to have complete control of all environmental conditions. The following parameters were used: 60% relative humidity, photosynthetically active radiation (PAR) of 250 µmol m^–2^ s^–1^ provided by a combination of fluorescent lamps (TL-D Master reflex 830 and 840, Koninklijke Philips Electronics N.V., the Netherlands) and high-pressure sodium lamps (Son-T Agro, Philips, Cambridge, MA, USA), and 16/8 h day/night with temperatures of 28/16 °C at first and then 43/30 °C during the short-term heat stress event. This experiment was performed at two temperatures CO_2_: 400 ppm CO_2_ and 1000 ppm CO_2_. The CO_2_ content inside the chamber was regulated by the injection of external compressed air or CO_2_, controlled by a Dräger Polytron IR CO_2_ detector. All plants of the study were drip-irrigated by self-compensating drippers (2 L h^−1^), always maintaining a drainage of 35%. After 86 days, half of the plants, which were randomly selected, received a foliar spraying with a solution containing 2.5 mM putrescine plus 0.01% Tween-20 as a surfactant. Each plant was fully sprayed with 20 mL of the putrescine solution by using a hand sprayer. The putrescine treatment was allowed to act for 4 days, and then half of the sprayed plants and half of control plants were collected. Those plants remaining in the climatic chamber were then exposed during 3 days to heat stress at 43/30 °C day/night. After that period, those plants were also collected.

Thus, this experiment concluded after 93 days after transplantation in the chamber.

### 2.2. Chemicals and Reagents

The following standards were acquired from Sigma-Aldrich (Steinheim, Germany): glucose, sucrose, fructose, inositol, spermidine, spermine, cadaverine, putrescine, 1,6-hexaendiamine gallic acid, 6-hydroxy-2,5,7,8-tetramethylchroman-2-carboxylic acid (Trolox), 2,2-azino-bis(3-ethylbenzothiazoline-6-sulphonic acid) diammonium salt (ABTS^•+^), and benzoyl chloride. Sodium hydroxide was purchased from Fluka (Buchs, Switzerland), and LC-MS grade organic solvents (Methanol, acetonitrile), together with sodium carbonate, Folin-Ciocalteu reagent, and ethyl ether, were purchased from Panreac Química (Barcelona, Spain). SPE cartridges (C18 Sep-Pak cartridges) were obtained from Waters Associates (Milford, MA, USA), and, lastly, ultrapure water was produced by a Millipore water purification system.

### 2.3. Growth Parameters Analysis

**Figure 1:**
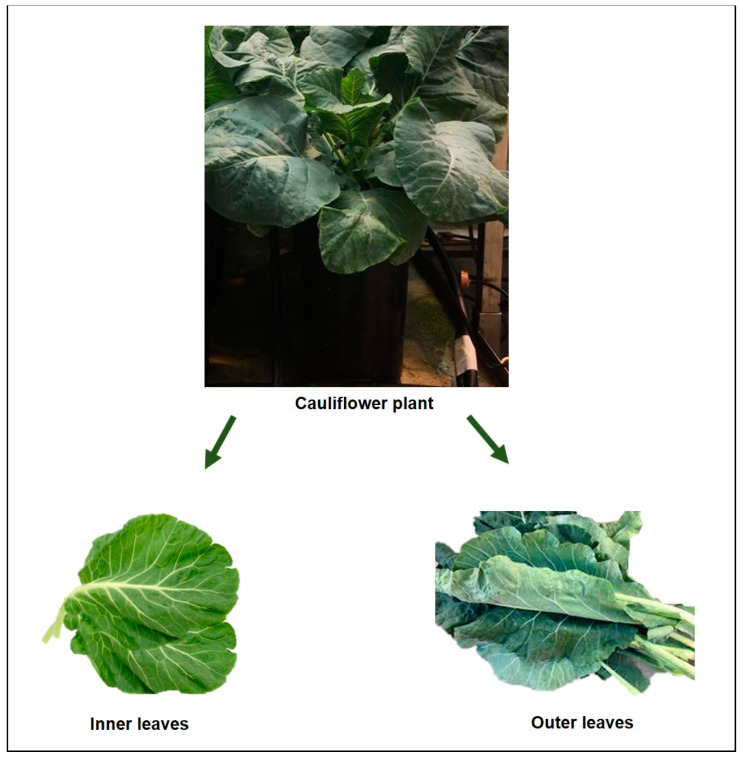
The physical characteristics of cauliflower.

Both the height of the cauliflower plants and the fresh weight of the leaves were determined on the final day of this experiment (the harvest day), and prior to their storage at −80 °C until analysis. The measurement of the height of the cauliflower was carried out using a meter. To measure the height, the longest leaf of the plant was taken into account. In order to safely and reliably determine the weight of the cauliflower leaves, each intact plant was placed in a collection tray and had its roots removed. After weighing the entire aerial part of the plant, the leaves of the inner and outer layers of the cauliflower were separated and weighed again. This leaf classification was performed according to a previous procedure of cauliflower leaf separation (Figure 1) [17].

### 2.4. Total Soluble Sugars Content Analysis

For their extraction and analysis, the methodology previously described by Balibrea et al. [18], with some modifications, was followed. In brief, 50 mg of inner and outer leaves was lyophilized and finely ground in a Moulinex grinder (AR110830); incubated twice with 1.5 mL of extraction buffer (MeOH/water (80:20, *v*/*v*) at 4 °C, for 30 min each time; and centrifuged for 15 min at 3500× *g*, at 4 °C. Then, each sample supernatant was filtered through an activated C18 Sep-Pak cartridge (Waters Associates, Milford, MA, USA) that had been previously activated. The activation of that cartridge was carried out by washing with 20 mL of methanol/water (80%/20%, *v*/*v*). Both filtrates were combined and filtered through a 0.45-μm filter (Millipore, Beford, MA, USA). The ion chromatography with an 817 Bioscan (Metrohm, Herisau, Switzerland) system equipped with a pulsed amperometric detector (PAD) and a gold electrode, using a METROHM Metrosep Carb 1–150 IC column (4.6 × 250 mm), which was heated to 32 °C, was used to identify and quantify the sugars present in these samples.

### 2.5. Determination of Free radical Scavenging Ability by the Use of ABTS^•+^ Radical Cation Assay and Assessment of the Total Phenolic Content (TPC)

The free radical scavenging activity of the freeze-dried outer and inner cauliflower leaf samples was determined by the ABTS radical cation (ABTS^•+^) assay, as previously described by Cano-Lamadrid et al. [19], with some modifications. In brief, samples (0.5 g) were sonicated with 10 mL of buffer (MeOH/water (80:20, *v*/*v*) + 1% HCl at room temperature) for 15 min and stored for 24 h at 4 °C. On the next day, the mixture was sonicated again under the aforementioned conditions and centrifuged at 10,000 rpm for 10 min at 10,000× *g*. Afterwards, 10 μL of each supernatant was mixed with 990 μL of the free radical solution, shaken, and placed in darkness for 10 min. The sample absorbance was read at 734 nm for the ABTS^+^* method. The results were expressed as µmol Trolox g^−1^ dry weight (DW).

The total polyphenol content (TPC) was analyzed using the Folin-Ciocâlteu colorimetric method following the procedure described by Kähkönen et al. [20], with slight modifications. 0.5 g of fresh outer and inner cauliflower leaf samples was homogenized with 5 mL of 80% acetone at room temperature and centrifuged at 10,000× *g*, at 4 °C for 10 min. Then, the 100 µL sample supernatant was mixed with 1 mL of the Folin–Ciocalteu reagent diluted with Milli-Q water (1:10), and 2 mL of Milli-Q water. Then, this mixture was stored at room temperature for 3 min and 2 mL of 20% sodium carbonate was added, shaken vigorously, and incubated for 1 h at room temperature in the dark. Then, sample absorbance was measured at 765 nm. The results were expressed as gallic acid equivalents (GAE), mg GAE 100 g^−1^ FW by using the standard curve prepared from authentic gallic acid. Both measurements (antioxidant activity and total phenolic content) were carried out in an ultraviolet-visible (UV-vis) spectrophotometer (Shimadzu CPS-240 model, Kyoto, Japan).

### 2.6. Determination and Quantification of Polyamines by UPLC-UV

The extraction of PAs from fresh samples was performed following the procedure described by Rodríguez et al. [21]. Briefly, the inner and outer leaves (5 g) were homogenized with 1.2 mL of solvent extractant (0.5% cold HClO_4_ (*v*/*v*)) for 1 min, and the mixture was centrifuged (Eppendorf centrifuge 5804R, Hamburg, Germany) at 12,000× *g* for 8 min, at 4 °C. Later, an aliquot of supernatant (500 µL) was derivatized with benzoyl chloride [9]; afterwards, this emulsion was mixed with 4 mL of saturated NaCl solution to stop the reaction and the final extraction of PAs, and, subsequently, 4 mL of cold diethyl ether was added to the mixture. This mixture was stored at −20 °C until all PAs were extracted to the organic phase. An aliquot (1.5 mL) of this organic phase was evaporated using a SpeedVac concentrator (Savant SPD121P, Thermo Scientific, Waltham, MA, USA). The residue of each sample was re-dissolved in 500 µL of the mobile phase (water/acetonitrile, 58/42%, *v*/*v*). Four PAs were analyzed by reverse phase chromatography using an ACQUITY UPLC system (Waters, Milford, MA, USA) coupled with a UV detector, as reported by Collado-Gonzalez et al. [9]. The chromatographic separation was carried out on a ACQUITY UPLC HSS T3 column (2.1 × 100 mm, 1.8 µm) (Waters Corp., Wexford, Ireland), the temperature of which was 40 °C. The solvent used as the mobile phase was a mixture of water and acetonitrile (58/42%, *v*/*v*). The injection volume was 10 μL, and the elution was performed at a flow rate of 0.55 mL min^–1^. Data acquisition and processing were performed using the Empower 2 (Waters) software, and polyamine peaks were detected at 254 nm. Five replicates per treatment were measured.

### 2.7. Statistical Analysis

The design of the experiments was completely randomized with five replications. The data were analyzed using SPSS software v.21 (IBM, Chicago, IL, USA). An analysis of variance was performed, and mean values were compared with a Tukey 0.05 test. The values for each replicate were averaged before the mean and standard error of each treatment were calculated. Combinations of different treatments were used—with two CO_2_ treatments (400 ppm and 1000 ppm), two temperatures (28 °C and 43 °C), and the presence/absence of 2.5 mM putrescine—with five plants per combination.

## 3. Results and Discussion

### 3.1. Analysis of Height and Biomass of Cauliflower Leaves

Our results show that the exposure of cauliflower to 43 °C affected the height of the plants. Raza et al., reported that effectively exposing plants to high temperatures could lead to lower plant height and cause accelerated senescence and lower plant productivity [22].

**Figure 2:**
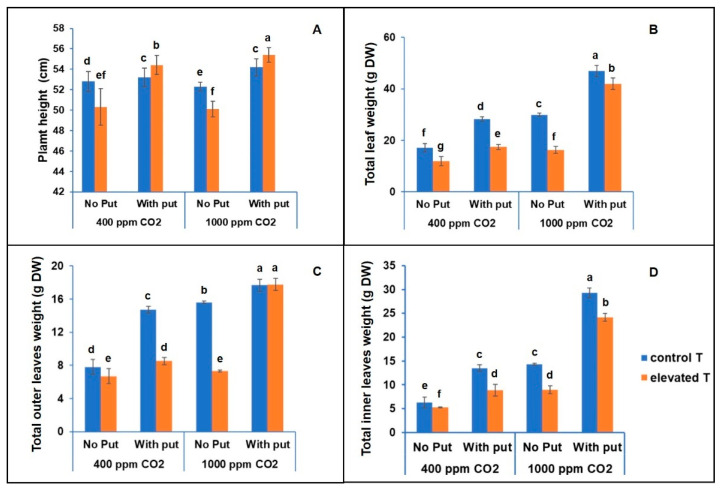
The effect of the foliar application of putrescine (2.5 mM) on the plant height (**A**), the total leaf weight FW (**B**), the total number of mature leaves (**C**), and the total number of inner leaves (**D**) of the cauliflower cv. Moonshine at different CO_2_ concentrations and temperatures. The data are presented as the treatment means (*n* = 5). Different small letters represent significantly different mean values according to Tukey’s test at *p* ≤ 0.05. Abbreviations used: No Put: in absence of putrescine; with Put: after using putrescine; control T (28 °C day/16 °C night): plants grown under control conditions; and elevated T: plants under heat stress (43 °C day/30 °C night).

Regarding the biomass, our results showed that cauliflower biomass ranged from 12.0 g to 28.3 g DW at control CO_2_ and from 16.3 to 29.9 g DW at elevated CO_2_. In the absence of short-term heat stress, the increase in CO_2_ led to a significant increase in plant biomass from 17.1 g to 47.0 g DW (1.8-fold), with a differential response observed after the short-term heat stress (Figure 2). The heat stress (43 °C), which lasted 3 days, led to a significant reduction in total leaf weight at both concentrations of CO_2_ from 17.1 to 12.0 g DW at control CO_2_ and from 29.9 to 16.3 g DW at elevated CO_2_), showing that under such heat conditions, the elevated CO_2_ increased biomass production by 20%, compared to the control CO_2_. Experimental evidence suggests that both the growth and the development of the plants in the *Brassicaceae* family begin to slow down when the temperature exceeds 28 °C [5]. In fact, Santos et al. [4] reported that excessive heat stress in broccoli crops was specifically harmful in the differentiation phase of flower buds. However, this damaging effect was reduced when heat stress was applied after the flower buds were already formed. The effect of temperature gradient on the growth of Brassicas depends mainly on several important factors, such as the crop and variety used, the exposure time, the intensity of stress, and the growth stages when heat stress is applied. [5]. Additionally, it has been reported that a high concentration of CO_2_ leads to an increase of the biomass of plants and chemical defenses [23].

Our data showed that the application of foliar putrescine at 2.5 mM to plants grown under elevated CO_2_ that were subjected to short-term heat stress also produced an increase from 16.3 g to 41.9 g DW. Thus, these conditions led to an increase of 2.6-fold of the biomass of plants with respect to those plants that were not treated with this polyamine (Figure 2). These results are consistent with those reported in other studies, where it was indicated that the foliar application of putrescine at appropriate concentrations can act as an elicitor for the improvement of the response of antioxidant enzymes in plants (superoxide dismutase (SOD) and catalase (CAT)) against abiotic stress [24]. The increased activities of these antioxidant enzymes induced the synthesis of some valuable metabolites, such as amino acids, soluble sugars, and proline. These metabolites may compensate against the adverse effects of abiotic stress in terms of both plant biomass and quality, and the quantity of certain bioactive compounds, such as monoterpenes [25].

### 3.2. Total Soluble Sugar Content

**Figure 3:**
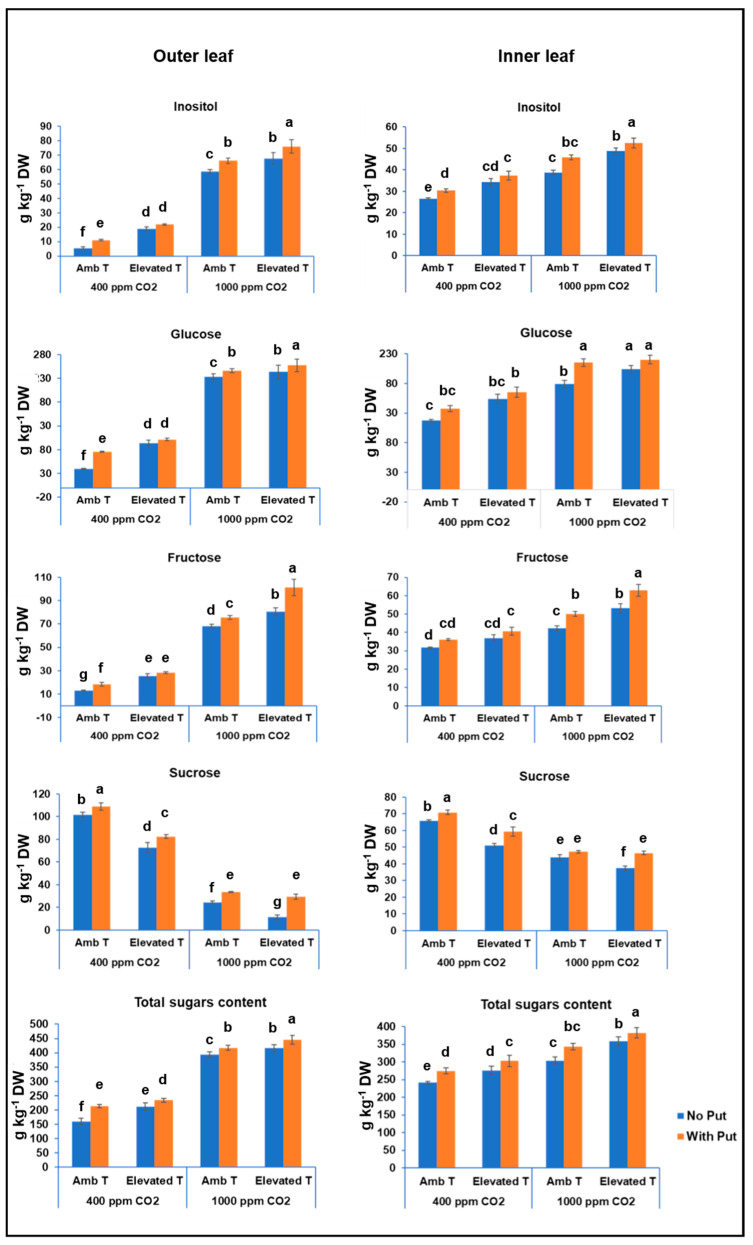
The effect of the foliar application of putrescine on the concentrations of sugars (g kg^−1^ DW) in outer and inner leaves of the cauliflower cv. Moonshine at different CO_2_ concentrations and temperatures. The data are presented as the treatment means (*n* = 5). Different small letters represent significantly different mean values according to Tukey’s test at *p* ≤ 0.05.

The main sugars in cauliflower leaves were inositol, glucose, fructose, and sucrose. As can be observed in Figure 3, the total sugar content oscillated between 159.6 and 445.8 g kg^−1^ DW, and between 241.0 and 382.1 g kg^−1^ DW for outer and inner leaves, respectively, with glucose being the predominant sugar in all cases. Bhandari and Kwak [26] studied the sugar content in the different tissues of three cauliflower varieties (Asia purple, Asia white, and Bridal) and of three broccoli varieties (AMaGi, Baeridom, and Cheonjae) and found that while sugar content was higher in broccoli florets than leaf tissue, in cauliflower the highest content was observed in leaf tissue. These authors also reported that sugar content varied from 214.3 to 241.6 and from 192.0 to 224.4 mg·g^−1^ DW for cauliflower and broccoli leaves, respectively. 

The sugar content found in the current work was higher than the sugar content in 20 cereals and 12 seed plants but was lower than the content found in twelve different fruits (from 201.6 g kg^−1^ DW (Korean cherry) to 387.7 g kg^−1^ DW (yellow peach). According to Guarise et al. [27], a higher content of sugars in leaves can be potentially associated with a longer useful plant life because plants can use sugars to maintain their basal metabolism. Furthermore, our data corroborate their affirmation that the foliar sugar content is associated with biomass production.

Under short-term heat stress conditions, sugar concentration significantly varied, with values between 210.9 g kg^−1^ DW and 445.8 g kg^−1^ DW in outer leaves and between 276.2 g kg^−1^ DW and 382.1 g kg^−1^ DW in inner leaves. This increase was a consequence of the increase of individual leaf sugar content. Especially, this was observed in the content of inositol (from 18.7 to 76.1 g kg^−1^ DW) and glucose (94.0 to 257.4 g kg^−1^ DW) in outer leaves. Their content was significant higher in treated plants (Figure 3 and Table 1). Nevertheless, this increase was influenced to a greater extent by the CO_2_ content than by short-term heat stress. This finding may be ascribed to the fact that exposure to a high CO_2_ content can suppress the photorespiration of plants, which results in the lower production of reactive oxygen species (ROS) and, hence, in a lower rate of oxidation. This underlines the stress-mitigating role of CO_2_ against the negative impact produced by abiotic stress. This is due to two types of factors: stomatal factors (greater stomatal closure and lower stomatal density) and non-stomatal factors. Among the non-stomatal factors, the changes in antioxidant enzymes (SOD, APX), the lower photorespiration rate, and an up-regulation of defense molecules should be mentioned [7]. In the absence of short-term heat stress at atmospheric CO_2_, the foliar putrescine application (2.5 mM) also resulted in a similar increase. 

Several authors have studied the direct effect of the foliar application of putrescine on plants and have reported that putrescine ameliorated the negative effect of heat stress on both the net photosynthetic rate and the chlorophyll content in leaves, resulting in an increase in the soluble sugar content in leaves [14,28]. As mentioned above, this could be due to putrescine acting to counteract the increase in reactive oxygen species, which is a consequence of the applied abiotic stress [25].

Regarding the age-dependent effect, it was observed that under high CO_2_, the range of sugar content varied between 393.6 and 445.8 g kg^−1^ DW in the mature leaves and between 303.9 and 382.1 g kg^−1^ DW in inner leaves. This may be due to the inner leaves lacking the natural ability to respond to stress suffered by the plant at high CO_2_ [29]. Moreover, it is necessary to take into account the fact that sugars not only participate in the regulation of a plant’s response to biotic and abiotic stresses but can also play an important role in the regulation of senescence [2]. Thus, a greater accumulation of soluble sugars at the beginning of senescence in different plant species has been found [30]. Previous studies have shown that high levels of CO_2_ generally lead to an accumulation of sugars and a decrease in nitrogen content, resulting an unbalanced C * N^−1^ ratio in outer leaves [31].

### 3.3. Antioxidant Activity

**Figure 4:**
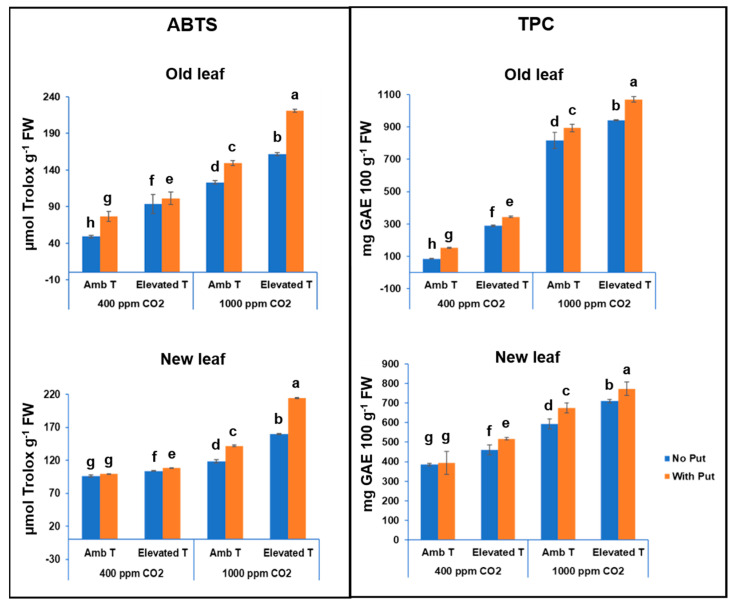
The antioxidant activity and total phenolic compounds in the outer and inner leaves of the cauliflower cv. Moonshine, along with the effect of short-term heat stress, CO_2_ concentration, and the foliar application of 2.5 mM putrescine. The data are presented as the treatment means (*n* = 5). Different letters represent significantly different mean values according to Tukey’s test at *p* ≤ 0.05.

Figure 4 shows that the antioxidant activity and the concentration of total phenolic compounds varied from 49.0 to 221.0 µmol Trolox g^−1^ DW and from 85.5 to 1070.2 mg GAE 100 g^−1^ FW for the outer leaves, and from 96.0 to 214.5 µmol Trolox g^−1^ DW and from 385.4 to 772.0 mg GAE 100 g^−1^ FW for the inner leaves. While many studies have focused on the study of antioxidant activity in the florets and reproductive organs of the *Brassicaceae* family, less attention has been paid to the effects of the sub-products on this plant family [32]. Our results showed lower antioxidant activity values in cauliflower by-products, compared to those observed in the edible part of the white cauliflower cv. Moonshine [9]. These values were found within the TPC range reported by other authors on the cauliflower by-products (533.6 mg GAE 100 g^−1^ DW and 914.1 mg GAE 100 g^−1^ DW) [26]. In comparison with other studies, our results could be considered to be richer in total polyphenol content than other vegetables and fruits, such as radish and apricots (79 mg GAE 100 g^−1^, both) [33]. This prompted us to think that cauliflower by-products could be considered to be a good source of TPC, and they may also possess good scavenging activity against ABTS^•+^ radicals. Although it is true that the antioxidant defense system of plants consists of enzymatic and non-enzymatic components, the antioxidant activity in crops of the *Brassicacea* family is mainly due to the content of phenolic compounds [34].

As can be observed in Figure 4, TPC and AA contents were also positively affected by the heat stress. In this sense, TPC varied between 288.8 and 1070.2 mg GAE 100 g^−1^ FW in outer leaves and between 459.8 and 772.0 mg GAE 100 g^−1^ FW in inner leaves, and AA varied from 93.5 to 221.1 µmol Trolox g^−1^ DW in outer leaves and from 103.7 to 214.5 µmol Trolox g^−1^ DW in inner leaves. The fact that short-term heat stress increases the content of polyphenols in cauliflower by-products (three-fold in outer leaves and 1.7-fold in inner leaves) and consequently their antioxidant activity (2.4-fold and 2-fold in outer and inner leaves, respectively) can be attributed to the greater adaptability of this crop. For example, Soengas et al. [34] stated that, as a defense mechanism against thermal stress, plants increased the biosynthesis of phenolic compounds by regulating their anabolism and catabolism. Consequently, a greater accumulation of phenolic compounds was observed.

Furthermore, as observed in Figure 4 and Table 1, the application of a high level of CO_2_ caused a sharper increase in antioxidant activity in plants subjected to short-term heat stress. This could be mainly due to the alleviating effect that CO_2_ has at elevated concentrations on the negative effects that abiotic stress has on cauliflower [7]. This was also observed in other plants (*Aster tripolium* L., *Lolium perenne* L., *Medicago sativa* L., *Quercus ilex* L., and *Arabidopsis thaliana*) subjected to various types of abiotic stresses (salinity, heat waves, and drought stress) and to CO_2_ [35,36,37]. 

Both TPC and AA in cauliflower leaves were also increased as a result of the exogenous application of putrescine. These results were to be expected since several previous works, including one carried out by our team, reported similar findings in several plants [25,38,39]. In such works, it was possible to see that the adverse effect produced in the plants by exposure to a high temperature could be mitigated by applying a foliar treatment with putrescine (2.5 mM).

As for leaf age, outer leaves contained a higher phenolic content and antioxidant activity. Similar results were observed by other authors, who reported that as a consequence of the imbalance of the C * N^−1^ ratio obtained in mature leaves from plants under elevated CO_2_, a premature senescence occurred in leaves. This is associated with a reduction in the activity of some antioxidant enzymes, which can increase oxidative stress and its subsequent higher content of polyphenols in these mature leaves [30,31].

### 3.4. Content of Polyamines

**Figure 5:**
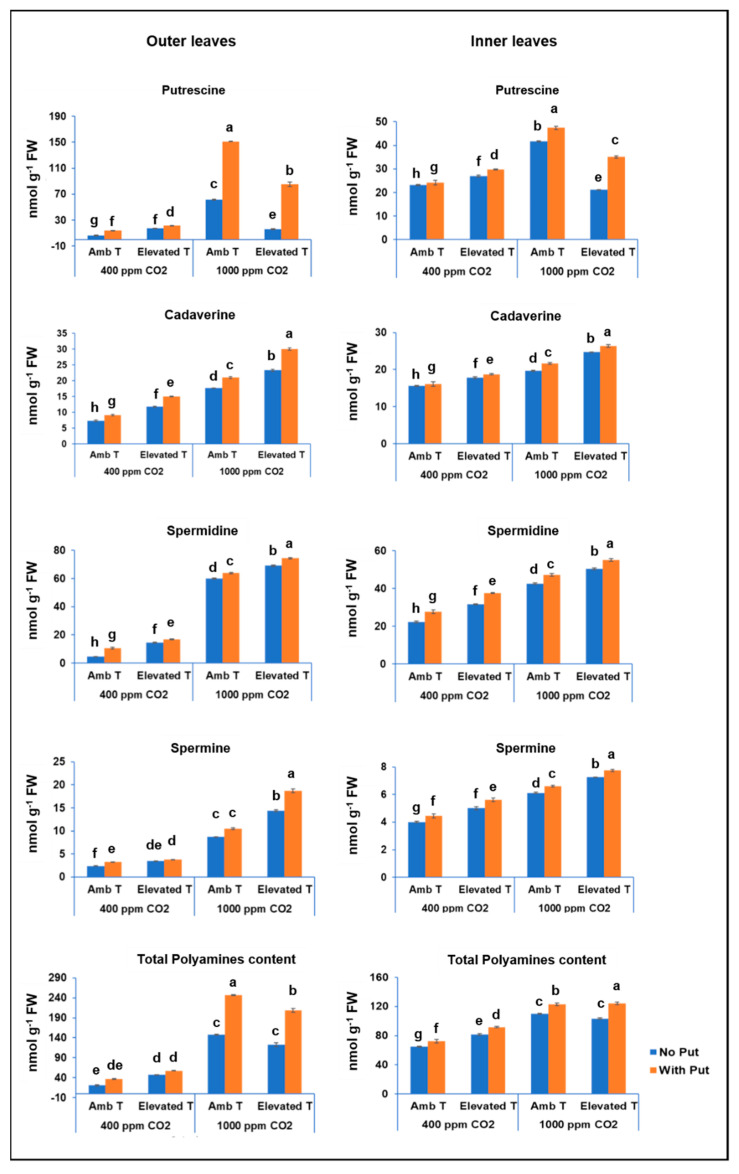
The effect of the foliar application of putrescine on the concentrations of polyamines (nmoles g^−1^) in the outer and inner leaves of the cauliflower cv. Moonshine at different CO_2_ concentrations and temperatures. The data are presented as the treatment means (*n* = 5). Different small letters represent significantly different mean values according to Tukey’s test at *p* ≤ 0.05.

Four free PAs, namely, putrescine, cadaverine, spermidine, and spermine, were found in the cauliflower by-products, with putrescine being the most abundant polyamine (Figure 5). As can be observed in Figure 5, the total polyamine content varied significantly between 20.5 and 208.6 nmoles g^−1^ FW for the outer leaves, and between 65.0 and 124.3 nmoles g^−1^ FW for the inner leaves.

The results reported here fit within the range reported for the content of PAs in different vegetables [11], prompting us to believe that these cauliflower by-products are rich sources of PAs. Therefore, even when compared to other plants within the *Brassicacea* family, it can be observed that our values obtained for cauliflower by-products were higher than those obtained for leaves of Brassica napus [40]. The beneficial effects on human health of the intake of the Brassica family of plants, in addition to being attributed only to the content of polyphenols [41], can also be attributed to other compounds such as polyamines. Some authors have indicated that there is a relationship between the intake of foods rich in PAs with a lower incidence of cardiovascular diseases, a lower rate of memory loss, and a lower reduced mortality [11]. Despite the fact that within the different types of abiotic stresses, heat stress is one of the two considered to be the most important, and despite the fact that PAs acted as indicators in plants for these different stresses, few studies have truly focused on the effect of heat stress on the content of PAs in plants. 

Data presented here showed a significant increase in endogenous free PAs content in response to short-term heat stress (Figure 5 and Table 1). In this sense, an increase of total free PAs from 20.5 to 46.9 nmoles g^−1^ FW in outer leaves, and from 65.0 until 81.6 nmoles g^−1^ FW in inner leaves, was observed as a response to short-term heat stress under atmospheric CO_2_. These findings are in agreement with the results found by Chen et al. [25] in a study carried out on Chinese kale. One possible reason for this increase is that PAs participate in maintaining the integrity of cell membranes under heat stress and play a crucial role in the adaptation of plants to heat stress [25]. Figure 5 and Table 1 also show that the combined effect of short-term heat stress and high CO_2_ on plants led to a higher concentration of free spermidine of 15.0% in outer leaves and of 18.2% in inner leaves, and of spermine of 65.8% in outer leaves and of 18.6% in inner leaves. However, it also resulted in an unexpected reduction in the concentration of free putrescine of 73.8% in outer leaves and of 50% in inner leaves. The results of the CO_2_ effect on polyamines were similar to those obtained in a study performed in tomato seedlings [42]. The reduction in putrescine content can be mainly attributed to the fact that an excessive accumulation of putrescine can generate significant damage to the lipids of the membrane and can even lead to an inhibition of plant growth [43]. At this point, it is worth mentioning that PAs can be found in their free form or conjugated with biological macromolecules such as nucleic acids and proteins by covalent bonds [44]. Thus, when plants are under abiotic conditions, their conjugated PAs become free PAs in order to sequester the ROS produced [45].

Furthermore, in this work we found an increase in all endogenous PAs content resulting from the application of foliar putrescine (2.5 mM) treatment (for putrescine: from 6.3 to 85.5 nmoles g^−1^ FW in the outer leaves and from 23.12 to 35.1 nmoles g^−1^ FW in the inner leaves; cadaverine: from 7.3 to 30.0 nmoles g^−1^ FW and from 15.6 to 26.3 nmoles g^−1^ FW in the outer and inner leaves, respectively; spermidine: from 4.5 to 74.4 nmoles g^−1^ FW and from 22.3 to 55.1 nmoles g^−1^ FW in the outer and inner leaves, respectively; and spermine: from 2.4 to 18.7 nmoles g^−1^ FW in the outer leaves and from 4.0 to 7.74 nmoles g^−1^ FW in the inner leaves, respectively) (Figure 5 and Table 1). This finding could be due to the fact that exogenous polyamine application can overcome the negative effects caused by abiotic conditions. This allows us to obtain a higher-quality plant and could even delay the senescence [25].

## 4. Conclusions

To sum up, the exposure of the plants to a high CO_2_ and the application of putrescine significantly promoted the accumulation of biomass (20%), total free sugar content (179% in outer leaves and 55% in inner leaves), total phenolic compounds (12- and 2-fold in the outer and inner leaves, respectively), and antioxidant activity (5 and 2-fold, in the outer and inner leaves, respectively) and total content of PAs (10- and 2-fold in the outer and inner leaves, respectively). These aspects improved the plants’ adaptability and allowed us to make the most out of the by-products of the cauliflower plants during short-term heat stress. The outer leaves of cauliflower cv. Moonshine grown under elevated CO_2_ and treated with putrescine, prior to exposure to high temperatures, showed the highest biosynthesis of sugars (20%), higher antioxidant activity (3%), and a higher content of total polyphenols (39%) and PAs (68%). Thus, these outer leaves could be considered as rich sources of polyphenols and PAs rather than by-products. These results indicate that this novel agronomic practice with putrescine could allow us to take advantage of both the continued increase in CO_2_, and the heat waves, which are becoming increasingly common. Moreover, these results should be taken into account to take advantage of these cauliflower by-products for pharmaceutical or nutraceutical purposes. Thus, these by-products could have a longer useful life, turning this crop into a more sustainable crop.

## Figures and Tables

**Table 1:** An ANOVA analysis of sugars, AA, TPC, and polyamines affected by putrescine treatment, the level of CO_2_, short-heat stress, and the age of leaves.

Attribute	T	Put	CO_2_	AL	T * Put	T * CO_2_	T * AL	Put * CO_2_	Put * AL	CO_2_ * AL	T * Put * CO_2_	T * Put * AL	T * CO_2_ * AL	Put * CO_2_ * AL	T * Put * CO_2_ * AL
Inositol	***	***	***	***	ns	ns	**	**	ns	***	ns	ns	ns	ns	ns
Glucose	***	***	***	***	***	***	ns	ns	ns	***	ns	ns	ns	*	*
Fructose	***	***	***	***	*	***	***	***	*	**	**	ns	ns	*	**
Sucrose	***	***	***	***	***	***	***	*	***	***	*	ns	***	*	ns
Total Sugars	***	***	***	***	ns	ns	ns	**	ns	***	ns	ns	ns	ns	**
AA	***	***	***	***	***	***	***	***	***	***	***	***	***	***	***
TPC	***	***	***	***	***	***	***	***	***	***	ns	ns	**	ns	***
Putrescine	***	***	***	***	***	***	***	***	***	***	***	***	***	***	***
Cadaverine	***	***	***	***	ns	ns	ns	*	ns	***	*	ns	ns	ns	**
Spermidine	***	***	***	***	*	ns	ns	**	ns	***	**	***	***	**	***
Spermine	***	***	***	***	***	***	***	***	***	***	***	***	***	***	***
Total PAs	***	***	***	***	***	ns	***		***	***	***	ns	***	***	**

The values are the means of five replicate samples; the means are within columns separated using Tukey’s multiple range test. *p* = 0.05; n.s.—non significant. *, **, and ***—significant at *p* ≤ 0.05, 0.005, and 0.001, respectively. Abbreviations used: AA: antioxidant activity; AL: age of leaf; CO_2_: level of CO_2_; T: temperature; PAs: polyamines; Put: putrescine; and TPC: total phenolic compounds.

## Data Availability

Data is contained within the article.

## References

[1] Aggarwal P., Vyas S., Thornton P., Campbell B., Kropff M. (2019). Importance of considering technology growth in impact assessments of climate change on agriculture. Glob. Food Secur..

[2] Zulfiqar F., Akram N.A., Ashraf M. (2020). Osmoprotection in plants under abiotic stresses: New insights into a classical phenomenon. Planta.

[3] Chaudhry S., Sidhu G.P.S. (2021). Climate change regulated abiotic stress mechanisms in plants: A comprehensive review. Plant Cell Rep..

[4] Santos A.R., Kano C., Maia Chaves F.C., Borborema da Cunha A.L., Felipe de Oliveira Gentil D., Batisrta da Costa Junior A. (2020). Inflorescence production of broccoli cultivars in the hot and humid climate of central amazonia. Rev. Caatinga.

[5] Ahmar S., Liaqat N., Hussain M., Salim M.A., Shabbir M.A., Ali M.Y., Noushahi H.A., Bilal M., Atta B., Rizwan M. (2019). Effect of abiotic stresses on *Brassica* Species and role of transgenic breeding for adaptation. Asian J. Crop Sci..

[6] Piñero M.C., Pérez-Jiménez M., López-Marín J., Del Amor F.M. (2017). Amelioration of boron toxicity in sweet pepper as affected by calcium management under an elevated CO_2_ concentration. Environ. Sci. Pollut. Res..

[7] Zinta G., AbdElgawad H., Peshev D., Weedon J.T., Van den Ende W., Nijs I., Janssens I.A., Beemster G.T., Asard H. (2018). Dynamics of metabolic responses to periods of combined heat and drought in Arabidopsis thaliana under ambient and elevated atmospheric CO_2_. J. Exp. Bot..

[8] Kapusta-Duch J., Szeląg-Sikora A., Sikora J., Niemiec M., Gródek-Szostak Z., Kuboń M., Leszczyńska T., Borczak B. (2019). Health-Promoting Properties of Fresh and Processed Purple Cauliflower. Sustainability.

[9] Collado-González J., Piñero M.C., Otálora G., López-Marín J., del Amor F.M. (2021). Exogenous spermidine modifies nutritional and bioactive constituents of cauliflower (*Brassica oleracea* var. botrytis L.) florets under heat stress. Sci. Hortic..

[10] Drabińska N., Jeż M., Nogueira M. (2021). Variation in the Accumulation of Phytochemicals and Their Bioactive Properties among the Aerial Parts of Cauliflower. Antioxidants.

[11] Muñoz-Esparza N.C., Latorre-Moratalla M.L., Comas-Basté O., Toro-Funes N., Veciana-Nogués M.T., Vidal-Carou M.C. (2019). Polyamines in Food. Front. Nutr..

[12] Ebert A.W. (2014). Potential of underutilized traditional vegetables and legume crops to contribute to food and nutritional security, income and more sustainable production systems. Sustainability.

[13] Nuñez-Gómez V., Baenas N., Navarro-González I., García-Alonso J., Moreno D.A., González-Barrio R., Periago-Castón M. (2020). Seasonal variation of health-promoting bioactives in broccoli and methyl-jasmonate pre-harvest treatments to enhance their contents. Foods.

[14] Collado-González J., Piñero M.C., Otálora G., López-Marín J., del Amor F.M. (2021). Effects of Different Nitrogen Forms and Exogenous Application of Putrescine on Heat Stress of Cauliflower: Photosynthetic Gas Exchange, Mineral Concentration and Lipid Peroxidation. Plants.

[15] Khedkar M.A., Nimbalkar P.R., Chavan P.V., Chendake Y.J., Bankar S.B. (2017). Cauliflower waste utilization for sustainable biobutanol production: Revelation of drying kinetics and bioprocess development. Bioproc. Biosyst. Eng..

[16] Del Amor F.M., Cuadra-Crespo P., Walker D.J., Cámara J.M., Madrid R. (2010). Effect of foliar application of antitranspirant on photosynthesis and water relations of pepper plants under different levels of CO_2_ and water stress. J. Plant Physiol..

[17] Saffeullah P., Siddiqi T.O., Umar S. (2021). Analysis of genetic, developmental and spatio-temporal patterns of nitrate accumulation in cauliflower and cabbage genotypes. Plant Physiol. Rep..

[18] Balibrea M.E., Cuartero J., Bolarín M.C., Pérez-Alfocea F. (2003). Sucrolytic activities during fruit development of Lycopersicon genotypes differing in tolerance to salinity. Physiol. Plant..

[19] Cano-Lamadrid M., Hernández F., Corell M., Burló F., Legua P., Moriana A., Carbonell-Barrachina Á.A. (2017). Antioxidant capacity, fatty acids profile, and descriptive sensory analysis of table olives as affected by deficit irrigation. J. Sci. Food Agric..

[20] Kähkönen M.P., Hopia A.I., Vuorela H.J., Rauha J.-P., Pihlaja K., Kujala T.S., Heinonen M. (1999). Antioxidant activity of plant extracts containing phenolic compounds. J. Agric. Food Chem..

[21] Rodriguez S., López B., Chaves A.R. (2001). Effect of different treatments on the evolution of polyamines during refrigerated storage of eggplants. J. Agric. Food Chem..

[22] Raza A., Razzaq A., Mehmood S.S., Zou X., Zhang X., Lv Y., Xu J. (2019). Impact of climate change on crops adaptation and strategies to tackle its outcome: A review. Plants.

[23] Fischer J.M. (2020). The Influences of Elevated Carbon Dioxide on Trichome Densities and Trichome Molecular Pathways. Doctoral Dissertation.

[24] Mohammadi H., Ghorbanpour M., Brestic M. (2018). Exogenous putrescine changes redox regulations and essential oil constituents in field-grown Thymus vulgaris L. under well-watered and drought stress conditions. Ind. Crops Prod..

[25] Chen D., Shao Q., Yin L., Younis A., Zheng B. (2019). Polyamine function in plants: Metabolism, regulation on development, and roles in abiotic stress responses. Front. Plant Sci..

[26] Bhandari S.R., Kwak J.-H. (2015). Chemical Composition and Antioxidant Activity in Different Tissues of *Brassica* Vegetables. Molecules.

[27] Guarise M., Borgonovo G., Bassoli A., Ferrante A. (2019). Evaluation of two wild populations of Hedge Mustard *(Sisymbrium officinale* (L.) Scop.) as a potential leafy vegetable. Horticulturae.

[28] Piñero M.C., Otálora G., Collado J., López-Marín J., Del Amor F.M. (2021). Foliar application of putrescine before a short-term heat stress improves the quality of melon fruits (*Cucumis melo* L.). J. Sci. Food Agric..

[29] Gamage D., Thompson M., Sutherland M., Hirotsu N., Makino A., Seneweera S. (2018). New insights into the cellular mechanisms of plant growth at elevated atmospheric carbon dioxide concentrations. Plant Cell Environ..

[30] Agüera E., De la Haba P. (2018). Leaf senescence in response to elevated atmospheric CO_2_ concentration and low nitrogen supply. Biol. Plant..

[31] Vicente R., Pérez P., Martínez-Carrasco R., Feil R., Lunn J.E., Watanabe M., Arrivault S., Stitt M., Hoefgen R., Morcuende R. (2016). Metabolic and transcriptional analysis of durum wheat responses to elevated CO_2_ at low and high nitrate supply. Plant Cell Physiol..

[32] Mageney V., Neugart S., Albach D.C. (2017). A guide to the variability of flavonoids in Brassica oleracea. Molecules.

[33] Cömert E.D., Mogol B.A., Gökmen V. (2020). Relationship between color and antioxidant capacity of fruits and vegetables. Curr. Res. Nutr. Food Sci..

[34] Soengas P., Rodríguez V.M., Velasco P., Cartea M.E. (2018). Effect of temperature stress on antioxidant defenses in *Brassica oleracea*. ACS Omega.

[35] Bhargava S., Mitra S. (2021). Elevated atmospheric CO_2_ and the future of crop plants. Plant Breed..

[36] Pintó-Marijuan M., Joffre R., Casals I., De Agazio M., Zacchini M., García-Plazaola J.I., Esteban R., Aranda X., Guàrdia M., Fleck I. (2013). Antioxidant and photoprotective responses to elevated CO_2_ and heat stress during holm oak regeneration by resprouting, evaluated with NIRS (near-infrared reflectance spectroscopy). Plant Biol..

[37] Zinta G., AbdElgawad H., Domagalska M.A., Vergauwen L., Knapen D., Nijs I., Janssens I.A., Beemster G.T., Asard H. (2014). Physiological, biochemical, and genome-wide transcriptional analysis reveals that elevated CO_2_ mitigates the impact of combined heat wave and drought stress in Arabidopsis thaliana at multiple organizational levels. Glob. Chang. Biol..

[38] Collado-González J., Piñero M.C., Otalora G., Lopez-Marín J., Del Amor F.M. (2022). Unraveling the nutritional and bioactive constituents in baby-leaf lettuce for challenging climate conditions. Food Chem..

[39] Mostafa H.A.M., Hassanein R.A., Khalil S.I., El-Khawas S.A., El-Bassiouny H.M.S., El-Monem A.A.A. (2010). Effect of arginine or putrescine on growth, yield and yield components of late sowing wheat. Res. J. Appl. Sci..

[40] Toscano S., Trivellini A., Cocetta G., Bulgari R., Francini A., Romano D., Ferrante A. (2019). Effect of Preharvest Abiotic Stresses on the Accumulation of Bioactive Compounds in Horticultural Produce. Front. Plant Sci..

[41] Martínez S., Armesto J., Gómez-Limia L., Carballo J. (2020). Impact of processing and storage on the nutritional and sensory properties and bioactive components of Brassica spp. A review. Food Chem..

[42] Zhang Y., Yao Q., Shi Y., Li X., Hou L., Xing G., Ahammed G.J. (2020). Elevated CO_2_ improves antioxidant capacity, ion homeostasis, and polyamine metabolism in tomato seedlings under Ca (NO_3_)^2^-induced salt stress. Sci. Hortic..

[43] Pál M., Szalai G., Janda T. (2015). Speculation: Polyamines are important in abiotic stress signaling. Plant Sci..

[44] Luna-Esquivel E.N., Ojeda-Barrios D.L., Guerrero-Prieto V.M., Ruiz-Anchondo T., Martínez-Téllez J.J. (2014). Poliaminas como indicadores de estrés en plantas. Rev. Chapingo Ser. Hortic..

[45] Park K.Y., Seo S.Y., Kim Y.J. (2019). Increasing polyamine contents enhances the stress tolerance via reinforcement of antioxidative properties. Front. Plant Sci..

